# Mieap-regulated mitochondrial quality control is frequently inactivated in human colorectal cancer

**DOI:** 10.1038/oncsis.2015.43

**Published:** 2016-01-04

**Authors:** H Kamino, Y Nakamura, M Tsuneki, H Sano, Y Miyamoto, N Kitamura, M Futamura, Y Kanai, H Taniguchi, D Shida, Y Kanemitsu, Y Moriya, K Yoshida, H Arakawa

**Affiliations:** 1 Division of Cancer Biology, National Cancer Center Research Institute, Tokyo, Japan; 2 Department of Surgical Oncology, Gifu University Graduate School of Medicine, Gifu, Japan; 3 Division of Molecular Pathology, National Cancer Center Research Institute, Tokyo, Japan; 4 Department of Pathology and Clinical Laboratories, National Cancer Center Hospital, Tokyo, Japan; 5 Department of Colorectal Surgery, National Cancer Center Hospital, Tokyo, Japan

## Abstract

Mieap, a p53-inducible protein, controls mitochondrial quality by repairing or eliminating unhealthy mitochondria. BNIP3 and NIX are critical mediators for the Mieap-regulated mitochondrial quality control. Mieap suppresses murine intestinal tumor via its mitochondrial quality control function. To explore the role of the Mieap-regulated mitochondria quality control function in colorectal cancer patients, we examined the statuses of p53, Mieap, BNIP3 and NIX in 57 primary colorectal cancer tissues. Promoter methylation of the *Mieap* and *BNIP3* genes was found in 9% and 47% of colorectal cancer cases, respectively, whereas p53 mutation was found in more than 50% of colorectal cancer tissues lacking methylation of the *Mieap* and *BNIP3* promoters, implying that the p53/Mieap/BNIP3-regulated mitochondria quality control pathway is inactivated in more than 70% of colorectal cancer patients. In LS174T colorectal cancer cells, hypoxia activated the Mieap-regulated mitochondria quality control function. Knockdown of p53, Mieap or BNIP3 in LS174T cells severely impaired the hypoxia-activated function, leading to the accumulation of unhealthy mitochondria and increase of mitochondrial reactive oxygen species generation. The mitochondrial reactive oxygen species generated by unhealthy mitochondria in the p53/Mieap/BNIP3-deficient cells remarkably enhanced cancer cell migration and invasion under hypoxic condition. These results suggest that the Mieap-regulated mitochondria quality control has a critical role in colorectal cancer suppression in the *in vivo* hypoxic tumor microenvironment.

## Introduction

Mieap, a p53-inducible protein, was recently identified as an important regulator of a novel mitochondrial quality control system.^1, 2, 3, 4^ We previously reported that Mieap regulates mitochondrial quality via two mechanisms.^1, 2^ One of the mechanisms, designated MALM (Mieap-induced Accumulation of Lysosomal proteins within Mitochondria), involves the repair of unhealthy mitochondria.^1^ In this mechanism, Mieap induces the accumulation of lysosomal proteins within the unhealthy mitochondria that produce high level of reactive oxygen species (ROS) to eliminate oxidized mitochondrial proteins.^1^ This process finally leads to a reduction of ROS generation and a restoration of mitochondrial adenosine triphosphate synthesis activity,^1^ thereby mediating the repair process of unhealthy mitochondria. Alternatively, the MIV (Mieap-induced vacuole) mechanism occurs,^2^ when MALM is blocked. In the MIV mechanism, Mieap induces a vacuole-like structure known as MIV in the cytoplasm.^2^ The MIV engulfs the severely damaged mitochondria and accumulates lysosomes, leading to the degradation of unhealthy mitochondria.^2^ This dynamic function of MIV is likely to represent atypical mitochondrial autophagy (mitophagy). Therefore, Mieap maintains mitochondrial integrity by repairing or eliminating unhealthy mitochondria via the MALM or MIV mechanism, respectively.^1, 2^

Previously, we reported that Bcl-2/adenovirus E1B 19 kDa interacting protein 3 (BNIP3) has a pivotal role in the Mieap-regulated mitochondrial quality control.^2, 3^ BNIP3 belongs to the Bcl-2 homology domain-3-only protein.^5^ It has been reported that BNIP3 expression is directly regulated by transcriptional factor HIF-1α under hypoxic conditions, via a hypoxia-response element located in the BNIP3 promoter.^6, 7, 8^ Forced expression of BNIP3 has been shown to induce cell death by inhibiting anti-apoptotic proteins, including Bcl-2 and Bcl-xL, but the BNIP3-induced cell death is neither inhibited by caspase inhibitors nor required a release of cytochrome c.^9, 10, 11^ However, our previous studies showed that enforced expression of exogenous BNIP3 does not induce cell death, whereas co-expression of exogenous Mieap, BNIP3 and NIX induces the opening of a pore in the mitochondrial double membrane, suggesting that BNIP3 may have a critical role in the translocation of lysosomal proteins from cytoplasm into mitochondria during the MALM process.^3^

To examine the role of Mieap in intestinal tumorigenesis *in vivo*, we recently established the Mieap-knockout mice and generated Mieap heterozygous (Apc^Min/+^Mieap^+/−^) and homozygous (Apc^Min/+^Mieap^−/−^) Apc^Min/+^ mice.^12^ Interestingly, the Apc^Min/+^ mice with the Mieap^+/−^ and Mieap^−/−^ genetic background exhibited markedly reduced lifespans compared with those of Apc^Min/+^ mice owing to the occurrence of severe anemia from intestinal bleeding.^12^ We further observed a substantial increase in the number and size of intestinal polyps in Mieap-deficient Apc^Min/+^ mice.^12^ Histopathologically, intestinal tumors in the Mieap-deficient Apc^Min/+^ mice clearly exhibited advanced grades of adenomas and adenocarcinomas.^12^ These results suggest that the Mieap-regulated mitochondrial quality control pathway has a critical role in the suppression of intestinal tumor *in vivo*.

Here, we report that inactivation of the Mieap-regulated mitochondrial quality control pathway occurs in more than 70% of colorectal cancer patients. *In vitro* experiments using LS174T human colon cancer cells indicated that hypoxia activates the Mieap-regulated mitochondrial quality control function, and that inactivation of the pathway promotes the accumulation of unhealthy mitochondria and increased mitochondrial ROS generation. The increased mitochondrial ROS in the p53/Mieap/BNIP3-deficient cells enhances cancer cell migration and invasion activity under hypoxia. These results suggest that the Mieap-regulated mitochondrial quality control pathway has a crucial role in tumor suppression in colorectal cancer patients.

## Results

### Frequent inactivation of Mieap-regulated mitochondrial quality control pathway in colorectal cancer patients

Previously, we reported that BNIP3 and NIX interact with Mieap in response to various artificial cellular stresses and mediate induction of the MALM, suggesting that BNIP3 and NIX have a critical role in the Mieap-regulated mitochondrial quality control pathway.^2, 3^ As Mieap expression is inactivated in more than 60% of cancer cell lines owing to the promoter methylation,^1^ we speculated that methylation of the *BNIP3* and/or *NIX* promoters could also be involved in the inactivation of the Mieap-regulated mitochondrial quality control pathway. Therefore, we examined the methylation of the *BNIP3* and *NIX* promoters in 21 cancer cell lines using methylation-specific PCR (MSP; Supplementary Figure S1). NIX promoter methylation was not found in any of the examined cell lines. However, the BNIP3 promoter was methylated in two colorectal cancer cell lines (SW480 and LoVo), suggesting that the BNIP3 gene is inactivated in colorectal cancer (Supplementary Figure S1).

**Figure 1:**
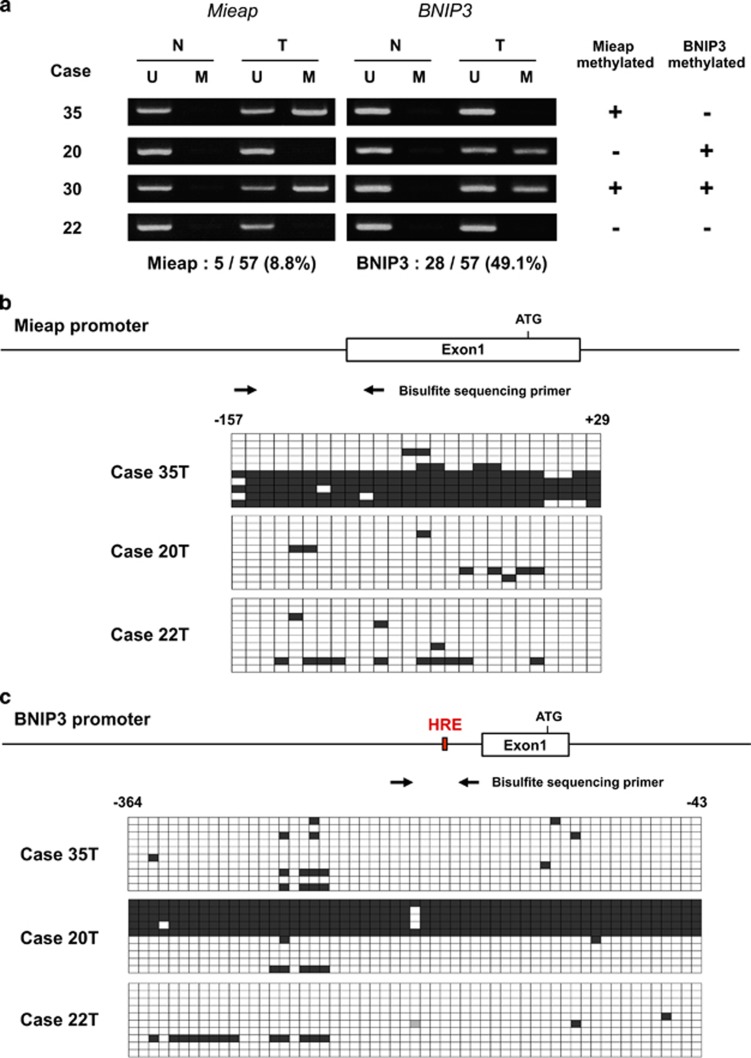
The promoter of Mieap and BNIP3 is frequently methylated in primary colorectal cancer tissues. (**a**) MSP analysis on the Mieap and BNIP3 promoter. The results of MSP on four representative cases 35, 20, 30 and 22 are shown. Each genomic DNA was extracted from primary colorectal tumor tissue (T) or corresponding normal colorectal tissue (N), and bisulfite-treated DNA was analyzed by MSP method. Mieap methylation was detected in 5/57 patients (8.8%) and BNIP3 methylation was detected in 28/58 patients (49.1%). All the samples from normal tissue did not show any Mieap and BNIP3 methylation. M, methylated; U, unmethylated. (**b** and **c**) Bisulfite sequencing on the Mieap and BNIP3 promoters. The results of bisulfite sequencing on the tumors of three representative cases 35, 20 and 22 T are shown. The promoters of Mieap (**b**) and BNIP3 (**c**) are examined by bisulfite sequencing using the indicated primers (black arrows). White square: unmethylated, black square: methylated. HRE, hypoxia-response element.

To explore the role of the Mieap-regulated mitochondrial quality control pathway in colorectal cancer, we examined the promoter-methylation status of the *Mieap*, *BNIP3* and *NIX* genes in primary cancer and corresponded normal tissues of 57 colorectal cancer patients by performing MSP. As shown in Figure 1a and Supplementary Table S1, Mieap promoter methylation was detected in 5 out of 57 patients (9%). Interestingly, BNIP3 promoter methylation was found in 28 out of 57 patients (47%, Figure 1a and Supplementary Table S1). Both the *BNIP3* and *Mieap* promoters were methylated in two patients. The more detailed methylation statuses of case 35, 20 and 22 T were confirmed using bisulfite-sequencing analysis (Figures 1b and c). The NIX promoter methylation was not detected in any of the 57 patients (0%). The Mieap and/or BNIP3 promoter methylation statuses are not correlated with any clinicopathological parameters on these patients (Supplementary Table S2).

The *Mieap* gene is a target of p53, and its expression is directly regulated by p53.^1^ Therefore, we reasoned that p53 mutations could contribute to the inactivation of the Mieap-regulated mitochondrial quality control pathway. Therefore, we examined the status of the *p53* gene in tumors from the 39 patients with (*n*=22) or without (*n*=17) *Mieap*/*BNIP3* promoter methylation by direct sequencing of the *p53* gene in these tumors. Interestingly, p53 mutation was found in 9 of the 17 patients without *Mieap*/*BNIP3* promoter methylation (52%) but only 4 of the 22 patients with *Mieap*/*BNIP3* promoter methylation (18%), suggesting that p53 is likely inactivated in colorectal cancers having normal Mieap and BNIP3 statues (Table 1).

Taken together, these results suggest that the p53/Mieap/BNIP3-regulated mitochondrial quality control pathway is inactivated in more than 70% of patients with primary colorectal cancer.

### Mieap-regulated mitochondrial quality control pathway is activated by hypoxic stress and inactivated by hemi-methylation of the BNIP3 promoter

The tumor microenvironment is hypoxic *in vivo*.^13^ We recently found that hypoxia induces the MALM in mouse and human normal cells, representing one of the two critical functions in the Mieap-regulated mitochondrial quality control (Nakamura, Tsuneki and Arakawa, manuscript in preparation). Under hypoxic conditions, HIF-1α activates the transcription of many target genes, including *BNIP3* and *NIX*, which are involved in hypoxic biological response.^6, 7, 8^ In addition, we previously demonstrated that Mieap interacts with BNIP3 and NIX in response to various genotoxic stresses including ionizing-irradiation and anticancer drug treatment in a ROS-dependent manner, suggesting that high level of ROS generated by unhealthy mitochondria have a key role in targeting unhealthy mitochondria via the interaction.^2, 3^ Therefore, we speculated that BNIP3 may mediate the Mieap-regulated mitochondrial quality control in hypoxic conditions by interacting with Mieap.

**Figure 2:**
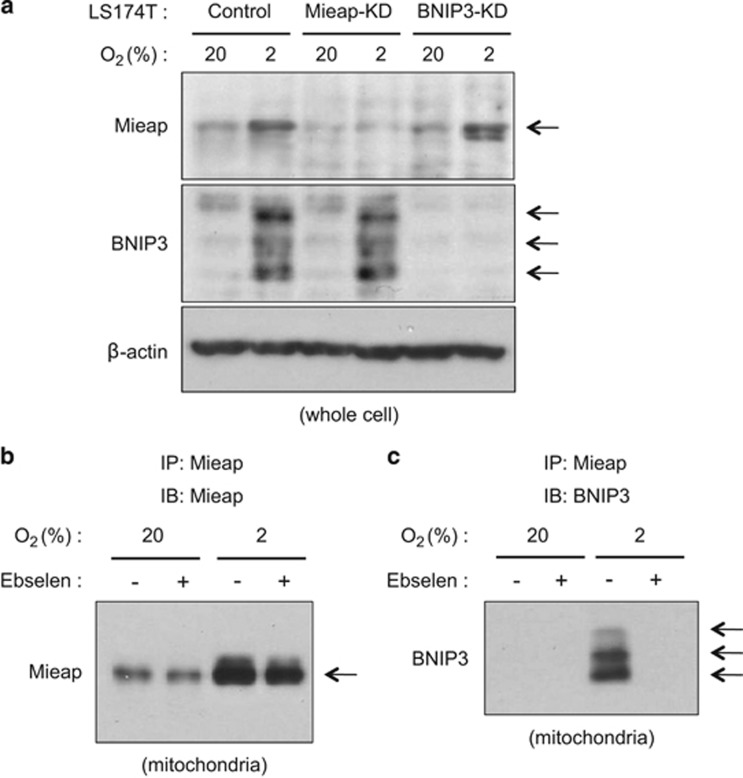
Mieap interacts with BNIP3 in response to hypoxia. (**a**) Induction of endogenous Mieap and BNIP3 proteins in response to hypoxia. LS174T Control, Mieap-KD and BNIP3-KD cells were cultured under 2% O_2_ hypoxia condition or 20% O_2_ normoxia condition for 48 h. Whole-cell lysate (whole cell) was used for SDS–PAGE, and each protein was detected by using anti-Mieap antibody or anti-BNIP3 antibody. β-Actin was used as loading control. (**b** and **c**) Interaction of endogenous Mieap and BNIP3 in response to hypoxia. The LS174T control cells were cultured under 2% O_2_ hypoxia condition or 20% O_2_ normoxia condition and in the presence (+) or absence (−) of Ebselen, and 2 days later, the cell lysates were purified from the mitochondrial fraction (mitochondria). The endogenous Mieap protein was precipitated from the lysates using an anti-Mieap antibody (**b**). The precipitated proteins were subjected to western blot analysis using an anti-Mieap antibody. The same blot as in **b** was subjected to immunoblotting using an anti-BNIP3 antibody (**c**). Arrows in **a**–**c** indicate Mieap protein band or BNIP3 protein bands. PAGE, polyacrylamide gel electrophoresis.

To evaluate this hypothesis, we examined whether hypoxia induces the expression of endogenous Mieap and BNIP3, and whether endogenous Mieap interacts with endogenous BNIP3 in response to hypoxic stress. As shown in Figure 2a, the expression of endogenous Mieap and BNIP3 proteins was increased in response to hypoxic stress. Moreover, endogenous Mieap actually interacted with endogenous BNIP3 under hypoxic conditions (Figures 2b and c). Interestingly, the ROS scavenger Ebselen canceled this interaction, implying that Mieap interacts with BNIP3 in a ROS-dependent manner (Figures 2b and c). Therefore, these results support our hypothesis that unhealthy mitochondria producing high level of ROS could be recognized by the Mieap-regulated mitochondrial quality control function through the interaction of the ROS-modified Mieap and BNIP3 proteins at the mitochondrial outer membrane in hypoxic conditions.^2, 3^

**Figure 3:**
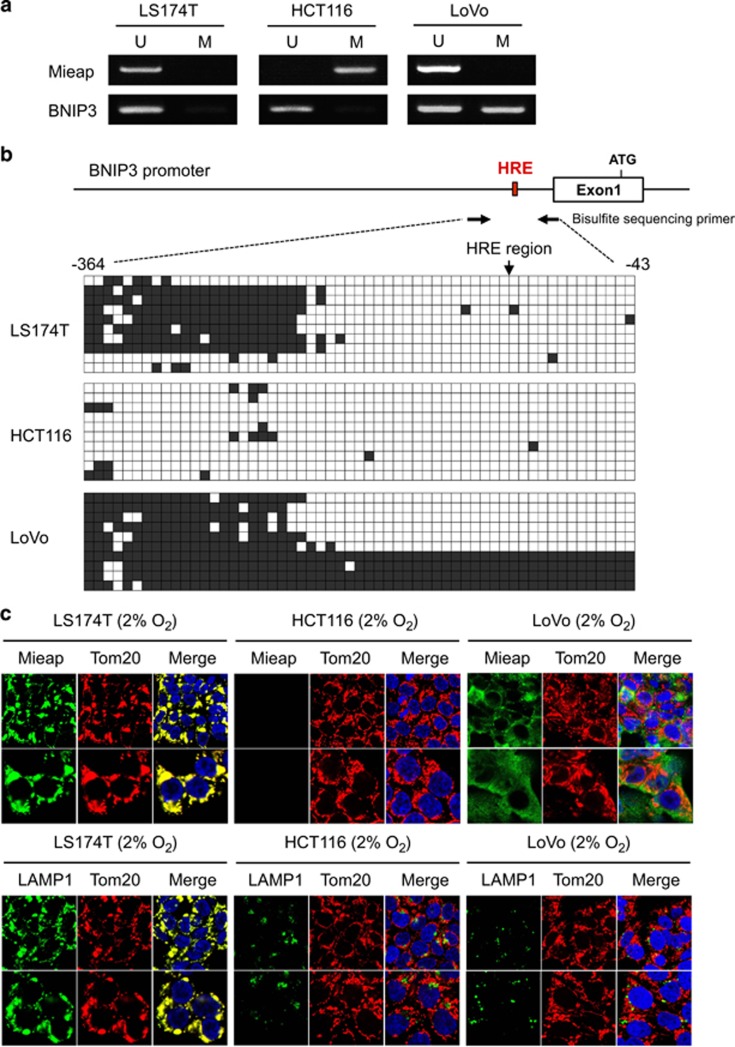
Mieap-regulated mitochondrial quality control is activated by hypoxia and severely inactivated by the BNIP3 promoter hemi-methylation. (**a**) MSP analysis of the Mieap and BNIP3 promoter on three colorectal cancer cell lines. Genomic DNAs were extracted from three colorectal cancer cell lines including LS174T, HCT116 and Lovo, and subjected to bisulfite treatment. MSP was performed using the MSP primer sets for Mieap and BNIP3. M, methylated; U, unmethylated. (**b**) Bisulfite sequencing analysis of the BNIP3 promoter on three colorectal cancer cell lines. The BNIP3 promoter is examined by bisulfite sequencing using the indicated primers (black arrows). White square: unmethylated, black square: methylated. HRE, hypoxia-response element. (**c**) Mieap-regulated mitochondrial quality control activity in three colorectal cancer cell lines under hypoxia. All three colorectal cancer cell lines were cultured under 2% O_2_ condition for 3 days, and then subjected to immunofluorescence (IF) experiment. Mieap protein was stained with polyclonal rabbit anti-Mieap antibody (Mieap: green). A lysosomal protein LAMP1 were stained with polyclonal rabbit anti-LAMP1 antibody (LAMP1: green). Mitochondria were stained with monoclonal mouse anti-TOM20 antibody (Tom20: red). The yellow areas indicated mitochondrial localization and accumulation of Mieap or LAMP1.

p53 and BNIP3 are two critical and indispensable mediators for the Mieap-regulated mitochondrial quality control function. Therefore, we hypothesized that inactivation of p53 and/or BNIP3 could contribute to a failure of the Mieap-regulated mitochondrial quality control function in hypoxic colorectal cancer tissues. Using three colorectal cancer cell lines (LS174T, HCT116 and Lovo), we examined this hypothesis *in vitro*. As shown in Figures 3a and b, although these three colorectal cancer cell lines retain wild-type p53, the promoters of the *Mieap* and *BNIP3* genes were methylated in HCT116 and Lovo cells, respectively. Therefore, we assumed that HCT116 and Lovo could be used to represent the Mieap-methyl (+)/BNIP3-methyl (−)/p53 wild-type phenotype and the Mieap-methyl (−)/BNIP3-methyl (+)/p53 wild-type phenotype, respectively (Figures 3a and b).

To evaluate the status of the Mieap-regulated mitochondrial quality control activity in these cells, we incubated the cells in hypoxic conditions for 3 days, and then carried out immunofluorescence experiments to assess MALM induction by hypoxia. As shown in Figure 3c and Supplementary Figure S2, the accumulation of the Mieap and LAMP1 proteins in the mitochondria was clearly detected in LS174T cells. However, LAMP1 was not accumulated in the mitochondria of HCT116 and Lovo cells. In particular, in Lovo cells, although a strong signal for Mieap was detected in the cytoplasm, Mieap and LAMP1 were not localized in the mitochondria (Figure 3c), confirming the critical role of BNIP3 in the MALM mechanism as we reported previously.^3^

### Knockdown of p53 or BNIP3 inactivates the Mieap-regulated mitochondrial quality control function and results in accumulation of unhealthy mitochondria producing high level of ROS

**Figure 4:**
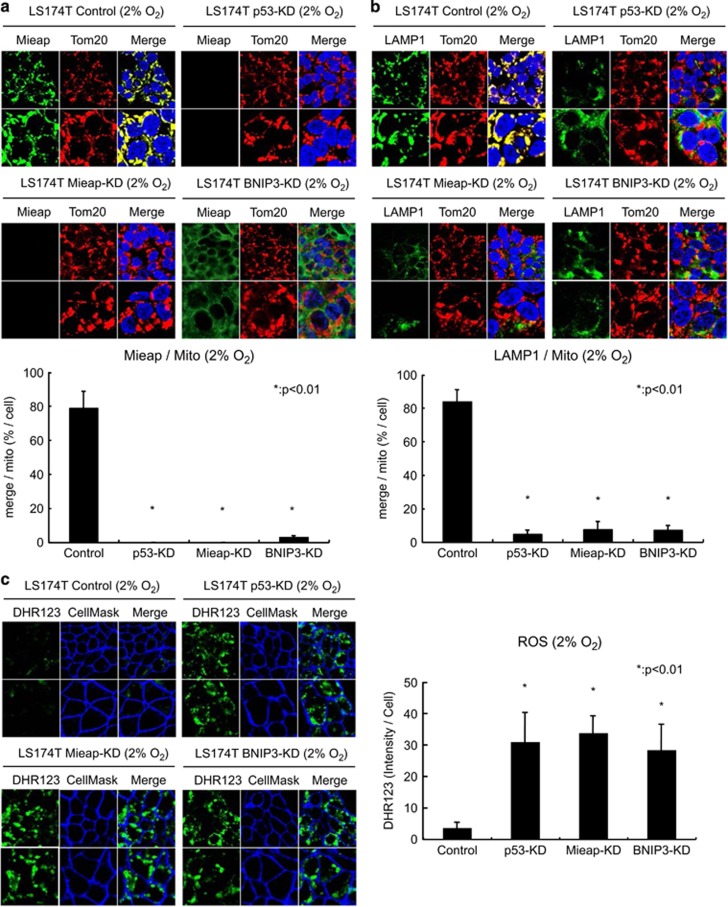
Knockdown of p53 or BNIP3 inhibits Mieap-regulated mitochondrial quality control under hypoxia and accumulates unhealthy mitochondria producing high level of ROS. (**a** and **b**) Mieap-regulated mitochondrial quality control activity in LS174T-cont, p53-KD, Mieap-KD and BNIP3-KD cells under hypoxia. All cell lines were cultured under 2% O_2_ condition for 3 days, and then subjected to immunofluorescence (IF) experiment (top). Mieap protein was stained with polyclonal rabbit anti-Mieap antibody (Mieap: green) (**a**). Lysosomes were stained with polyclonal rabbit anti-LAMP1 antibody (LAMP1: green) (**b**). Mitochondria were stained with monoclonal mouse anti-TOM20 antibody (Tom20: red). The yellow areas indicated mitochondrial localization and accumulation of Mieap (**a**) or LAMP1 (**b**). Quantitative analysis of the yellow or red areas was carried out in 300–400 cells (bottom). Average values for the ratio of yellow to red signals (merged/mitochondrial; bar graph) are presented; the error bars indicate 1 s.d.; *P<0.01 was considered statistically significant. (**c**) Mitochondrial ROS in LS174T-cont, p53-KD, Mieap-KD and BNIP3-KD cells under hypoxia. All cell lines were cultured under 2% O_2_ condition for 3 days, and then examined on mitochondrial ROS level by staining with DHR123 (green). Cells were also stained with CellMask (blue) to count the number of cells. Quantitative analysis of ROS was carried out in 300–400 cells. Average intensities of ROS per cell are shown with error bars indicating 1 s.d.; *P<0.01 was considered statistically significant.

To further confirm the roles of p53, Mieap and BNIP3 in the Mieap-regulated mitochondrial quality control function in colorectal cancer, we prepared the p53-knockdown (KD), Mieap-KD and BNIP3-KD cells using LS174T cells. As shown in Figures 4a and b, Mieap and LAMP1 proteins accumulated in the mitochondria of LS174T control cells under hypoxic conditions. However, deficiencies in p53, Mieap or BNIP3 completely inhibited the hypoxia-induced MALM. These results, taken together, suggest that p53 and BNIP3 have an indispensable role in the Mieap-regulated mitochondrial quality control function in colorectal cancer cells under hypoxic conditions.

We previously reported that the unhealthy mitochondria in MALM-deficient cells produce high level of mitochondrial ROS in response to various stresses, including ionizing irradiation, genotoxic agent and H_2_O_2_.^1,2^ Therefore, we next examined whether the mitochondria in the MALM-deficient colorectal cancer cells generate high levels of ROS under hypoxia. As shown in Figure 4c, the mitochondria in LS174T-p53-KD, Mieap-KD and BNIP3-KD cells produced high levels of ROS under hypoxia, whereas only a small amount of ROS were detected in the mitochondria of LS174T control cells, implying that the unhealthy mitochondria accumulate in the MALM-deficient colorectal cancer cells and produce high level of ROS under hypoxic conditions.

### Inactivation of the Mieap-regulated mitochondrial quality control pathway enhances colorectal cancer cell migration and invasion through ROS generation by unhealthy mitochondria

The accumulation of unhealthy mitochondria in MALM-deficient colorectal cancer cells could be advantageous to the tumorigenesis of colorectal cancer. As mitochondrial ROS have previously been shown to have a critical role in cancer metastasis,^14^ we examined the role of ROS generation by unhealthy mitochondria that accumulated in MALM-deficient colorectal cancer cells.

**Figure 5:**
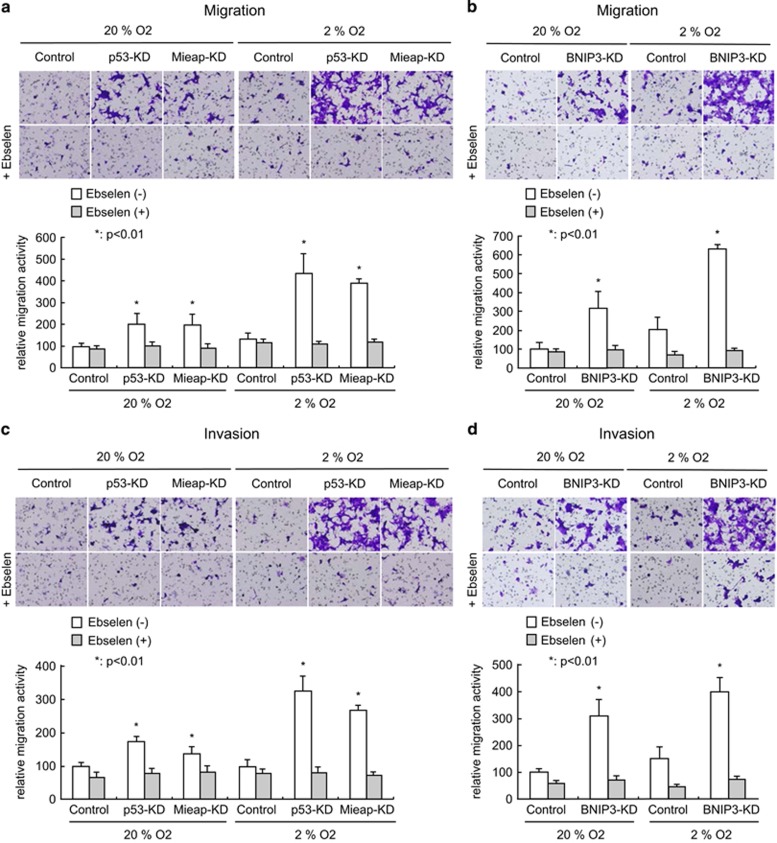
Inactivation of Mieap-regulated mitochondrial quality control upregulates colorectal cancer cell migration and invasion via unhealthy mitochondria-produced ROS. (**a** and **b**) Cell migration analysis of LS174T control, p53-KD, Mieap-KD and BNIP3-KD cells. (**c** and **d**) Cell invasion analysis of LS174T control, p53-KD, Mieap-KD and BNIP3-KD cells. All cell lines were cultured on collagen-coated chamber (for cell migration) or on Matrigel-treated chamber (for cell invasion) under 2% O_2_ hypoxia or 20% normoxia condition with or without 50 μm Ebselen for 2 days, and then cells were fixed, stained with Crystal Violet and subjected to the quantification study. Migrated cells (**a** and **b**) or invaded cells (**c** and **d**) were fixed and visualized by staining of Crystal Violet (upper panel), and relative migration activity was quantified as bar graph (lower panel). The relative intensity of samples were shown relative to those of corresponding intensity in LS174T Control/20% O_2_/Ebselen (−) sample as 100. Average intensities of stained cell per photo are shown with error bars indicating 1 s.d.; *P<0.01 was considered statistically significant.

Therefore, we examined cell migration and invasion activities in MALM-inducible (LS174T control) and MALM-deficient (LS174T-p53-KD and Mieap-KD) colorectal cancer cells under normoxic and hypoxic conditions. Both of the MALM-deficient colorectal cancer cells (LS174T-p53-KD and Mieap-KD) exhibited significantly increased cell migration and invasion, even under normoxic conditions, as compared with that of MALM-inducible LS174T control cells (Figures 5a and c). Interestingly, the cell migration and invasion activities in MALM-deficient LS174T-p53-KD and Mieap-KD cells were dramatically upregulated after exposure to hypoxia (Figures 5a and c). Those increased activities were completely inhibited by treatment with the ROS scavenger Ebselen (Figures 5a and c). These results were further confirmed in MALM-deficient LS174T-BNIP3-KD cells (Figures 5b and d).

Thus, taken together, these results suggest that inactivation of the p53/Mieap/BNIP3-regulated mitochondrial quality control pathway results in the accumulation of unhealthy mitochondria in colorectal cancer cells; these unhealthy mitochondria produce high level of ROS under hypoxic conditions, and ROS generated by the unhealthy mitochondria in MALM-deficient colorectal cancer cells could contribute to cancer invasion and metastasis.

## Discussion

In our current study, we found that inactivation of p53/Mieap/BNIP3 pathway occurred in more than 70% of patients with colorectal cancer. This result strongly suggests that this pathway has a critical role in suppressing colorectal cancer. Previous studies have reported that DNA methylation of the *BNIP3* promoter occurs frequently in pancreatic, gastric and colorectal primary tumors and cell lines.^15, 16, 17, 18^ However, the role of BNIP3 inactivation in these cancers remains unclear. BNIP3 is thought to induce necrotic-like cell death through the opening of the mitochondrial permeability transition pore without the release of cytochrome c or caspase activation.^9, 10, 11, 19, 20^ In addition, BNIP3 can be induced under hypoxic stress as a target gene of HIF-1a, and has been shown to be involved in hypoxia-induced cell death.^5, 11^ Therefore, methylation of the *BNIP3* promoter was thought to contribute to cell death-resistant phenotype.

However, we recently demonstrated a new role of BNIP3 in mitochondrial quality control.^3^ Mieap binds to BNIP3 via the BH3 domain of BNIP3 and the coiled-coil domains of Mieap in a ROS-dependent manner.^3^ The interaction among Mieap, BNIP3 and NIX is able to induce the MALM mechanism to repair unhealthy mitochondria.^2, 3^ Conversely, deficiencies in MALM induction lead to the accumulation of unhealthy mitochondria generating high level of ROS. Therefore, methylation of the *BNIP3* promoter in primary colon cancer may contribute to the impairment of MALM induction *in vivo*, leading to failure of the Mieap-regulated mitochondrial quality control function and accumulation of unhealthy mitochondria generating high levels of ROS in the tumor microenvironment. In fact, in this study, we showed that two colorectal cancer cell lines, HCT116 [p53 wt, Mieap-methyl (+), BNIP3-methyl (−)] and Lovo [p53 wt, Mieap-methyl (−), BNIP3-methyl (+)], failed to induce MALM in response to hypoxic stress. In addition, we confirmed that the hypoxia-induced MALM mechanism was severely defective in p53-KD, Mieap-KD and BNIP3-KD LS174T cells, in which the unhealthy mitochondria accumulated and produced high levels of ROS. Therefore, these results suggest that the Mieap-regulated mitochondrial quality control function is frequently lost in primary colorectal cancer tissues, in which unhealthy mitochondria accumulate and produce high levels of ROS in the hypoxic tumor microenvironment.

Recently, we found that Mieap-deficient Apc^Min/+^ mice exhibited a robust increase of intestinal tumors and remarkable advanced grade of adenomas and adenocarcinomas.^12^ These results clearly suggest that Mieap is involved in intestinal tumorigenesis *in vivo*. Furthermore, we demonstrated that unhealthy mitochondria dramatically accumulate in normal intestinal epithelial cells and tumor cells in Mieap-deficient Apc^Min/+^ mice, leading to increased oxidative stresses in these Mieap-deficient cells.^12^ Consistent with previous observation,^21^ our data suggest that accumulation of unhealthy mitochondria and increased ROS generation by such unhealthy mitochondria may accelerate intestinal tumor initiation and progression *in vivo*. Therefore, the findings obtained from the tumor tissues of clinical colorectal cancer patients and intestinal tumor animal models strongly support our hypothesis that the Mieap-regulated mitochondrial quality control has a crucial role in colorectal tumor suppression *in vivo* by controlling mitochondrial oxidative stress.

**Figure 6:**
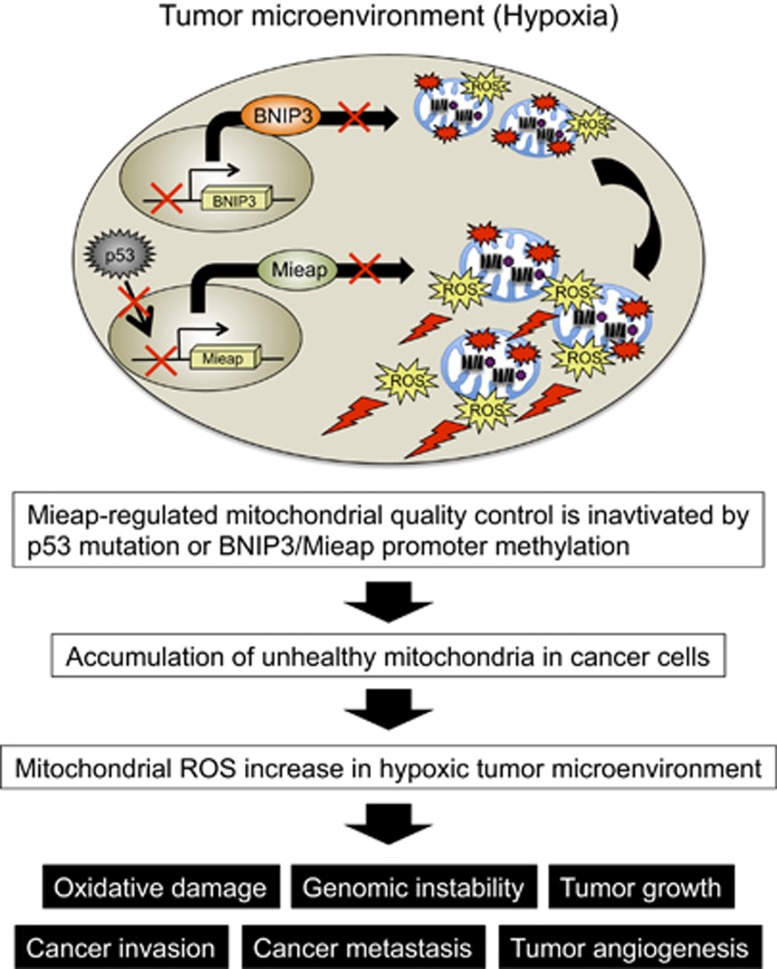
Hypothetical model for alteration of Mieap-regulated mitochondrial quality control in cancer. Tumor microenvironment is hypoxic. In hypoxic tumor microenvironment, the Mieap-regulated mitochondrial quality control is frequently inactivated by p53 mutation or the Mieap/BNIP3 promoter methylation in colorectal cancer cells. Therefore, unhealthy mitochondria accumulate in cancer cells and produce high level of ROS under hypoxia. The elevated mitochondrial ROS causes oxidative damage to DNA, RNA, protein and lipid and so on. This may induce genomic instability. The mitochondrial ROS contribute to tumor growth, epithelial–mesenchymal transition, cancer invasion, cancer metastasis, tumor angiogenesis through the activation of HIF-1, NF-κB, MMPs, AKT, Erk1/2, JNK and so on. We propose that the Mieap-regulated mitochondrial quality control is a tumor suppressor for colorectal cancer. Erk, extracellular signal-regulated kinase; MMP, matrix metalloprotease; NF-κB, nuclear factor kappaB.

Compared with normal cells, many types of cancer cells have increased levels of ROS.^22, 23^ Mitochondrial ROS are closely associated with activation of matrix metalloproteases.^24^ Mitochondrial ROS stabilize and activate HIF-1α under hypoxic environment, leading to the promotion of tumor angiogenesis and growth.^25, 26, 27^ Mitochondrial ROS also activate nuclear factor kappaB (NF-κB), which regulates cell survival and growth pathways.^28^ Increased ROS level is involved in the upregulation of cell proliferation via activation of the PI3K/Akt pathway or the mitogen-activated protein kinase/extracellular signal-regulated kinase 1/2 pathway.^29, 30, 31, 32^ Mitochondrial ROS and hypoxia are involved in induction of epithelial–mesenchymal transition, which has a critical role in cancer invasion and metastasis.^33, 34, 35^ Finally, Ishikawa *et al.*^14^ clearly demonstrated that increased levels of mitochondrial ROS due to a mitochondria DNA mutation could contribute to tumor progression by enhancing the metastatic potential of tumor cells. All of these data strongly suggest that mitochondrial ROS has a very important role in cancer progression and metastasis in the tumor microenvironment. Therefore, these previous observations and our results in this study strongly support our hypothesis that inactivation of the Mieap-regulated mitochondrial quality control pathway leads to the accumulation of unhealthy mitochondria generating high level of ROS in the tumor microenvironment and thereby promoting cancer cell growth, migration, invasion and metastasis *in vivo* (Figure 6).

As almost half of the 57 colorectal cancer patients enrolled in the present study exhibited *BNIP3* promoter methylation, we speculate that inactivation of the *BNIP3* gene may occur during the early stage of colon cancer development. Interestingly, our previous studies demonstrated that knockdown of NIX or BNIP3 severely impairs MALM induction, but strongly induces MIV generation.^2, 3^ MIV has a critical role in the elimination of the severely damaged mitochondria as a function of mitophagy. Therefore, it is possible that tumors with only *BNIP3* promoter methylation may promote MIV generation, as previously seen in BNIP3-KD or NIX-KD cells.^2, 3^ In addition, as shown in Figure 4c, the mitochondria in LS174T BNIP3-KD cells produced high levels of ROS, which may enhance MIV generation. Therefore, in this case, the tumor may tend to suppress MIV generation by inactivating *p53* or *Mieap* via a *p53* mutation or *Mieap* promoter methylation, respectively. In fact, we found two cases showing both *BNIP3* and *Mieap* promoter methylation. Therefore, we speculate that *BNIP3* promoter methylation occurs earlier than *Mieap* promoter methylation, and that BNIP3 inactivation causes accumulation of ROS-generating mitochondria, leading to the enhanced generation of MIV. This may explain why Mieap must be inactivated in BNIP3-deficient cells.

In summary, in this study, we found that the Mieap-regulated mitochondrial quality control pathway is frequently inactivated in primary tumors of colorectal patients. Inactivation of this function leads to accumulation of unhealthy mitochondria and excessive production of mitochondrial ROS under hypoxic conditions, which contributes to promotion of cancer cell migration and invasion (Figure 6). Further investigation on the Mieap-regulated mitochondrial quality control system via two critical functions, MALM and MIV, should provide a deeper understanding of the role of unhealthy mitochondria and mitochondrial ROS in *in vivo* intestinal tumorigenesis. As unhealthy mitochondria and high levels of mitochondrial ROS are considered specific characteristics of cancer cells and a driving force behind cancer development, our findings suggest that inhibition of cancer mitochondria and/or mitochondrial ROS may represent a new strategy for cancer treatment in the future.

## Materials and methods

### Primary colorectal tumors

All primary colorectal tumor samples and corresponding normal tissues were obtained from the surgical specimens of 57 patients treated at the National Cancer Center Hospital in Tokyo, Japan. Samples were frozen immediately in liquid nitrogen and stored at −80 °C until used. The tissues and clinical information used in this study were obtained under informed consent. All experimental protocols were approved by the National Cancer Center Institutional Review Board.

### Cell lines

The following human cancer cell lines were used and purchased from the American Type Culture Collection (Manassas, VA, USA): LS174T, HCT116, HT-29, SW480 and LoVo (colorectal adenocarcinoma); HepG2 (hepatoblastoma); A549 and H1299 (lung cancer); MCF7 and T47D (mammary carcinoma); SK-N-AS (neuroblastoma); U87MG, U138MG, U373MG and T98G (glioblastoma); HeLa (cervical cancer); SaOS2 (osteosarcoma); Tera2 (malignant embryonal carcinoma) and TE-1 and TE-2 (esophageal cancer). The TERT-immortalized normal cell line HFF2 (human fibroblast cell) was provided by T Kiyono (National Cancer Center Research Institute, Japan) and DA Galloway (Fred Hutchinson Cancer Research Center, USA). The cells were cultured under the conditions recommended by their depositors.

### MSP and bisulfite sequencing

Genomic DNAs were extracted by using QIAGEN DNeasy blood and tissue kit from culture cells, and genomic DNA and total RNA were extracted by using QIAGEN Allprep DNA/RNA kit from colon tumor tissues (QIAGEN, Hilden, Germany). Each protocol was performed according to the user's manual. Bisulfite treatment of DNA, MSP and bisulfite sequencing were performed as described previously.^1^ The MSP reaction for Mieap was carried out as described previously,^1^ and the MSP reaction for BNIP3 was carried out using the following conditions: one cycle at 94 °C for 5 min, 35 cycles at 94 °C for 30 s, 57 °C for 30 s and 72 °C for 1 min, and final extension step at 72 °C for 7 min. BNIP3 primer sequences were, for M-set: forward, 5′-ACGCGTCGTACGTGTTATAC-3′ and reverse, 5′-AACTACGCTCCCGAACTAAA-3′ for UM-set: forward, 5′-GTATGTGTTGTATGTGTTATAT-3′ and reverse, 5′-CCAACTACACTCCCAAACTAAA-3′.

Bisulfite sequencing was performed with the common to methylated and unmethylated DNA sequences, using 1 μl sodium bisulfite-treated DNA from culture cells or colon tumors. The PCR reaction was carried out using the following conditions: one cycle at 94 °C for 5 min, 35 cycles at 94 °C for 30 s, 55 °C (Mieap) or 58 °C (BNIP3) for 30 s, and 72 °C for 1 min, and final extension step at 72 °C for 7 min. The PCR product was cloned into the TOPO-TA vector pCR2.1 (Invitrogen, Carlsbad, CA, USA). In total, 10 subclones were confirmed by restriction analysis and sequenced using the M13 forward primer (Invitrogen) with an ABI 3100 genetic analyser (Applied Biosystems, Life Technologies, Paisley, UK). The sequences of Mieap and BNIP3 primer sets for bisulfite sequencing were described in previous study.^1, 16^

### Antibody

The anti-Mieap antibody was prepared as described previously.^1^ The other primary antibodies used in this study were mouse monoclonal anti-TOM20 antibody (sc-17764, Santa Cruz Biotechnology, Santa Cruz, CA, USA), rabbit polyclonal anti-LAMP1 antibody (sc-5570, Santa Cruz), mouse monoclonal anti-BNIP3 antibody (ANa40, Abcam, Cambridge, UK) and mouse monoclonal anti-β-actin antibody (clone AC-74, Sigma-Aldrich, St Louis, MO, USA).

### Immunoprecipitation

To examine the interaction of endogenous Mieap and endogenous BNIP3, LS174T cont cells were incubated in an airtight modulator incubator (TAITEC, Saitama, Japan) with hypoxic gas (2% O_2_, 5% CO_2_ and 93% N_2_) or normoxic gas (20% O_2_, and 5% CO_2_) for 48 h in the presence or absence of Ebselen, and then the mitochondria were fractionated as previously described.^1^ The mitochondrial pellets were lysed on ice for 15 min in 500 μl of NP40 lysis buffer (1% NP40, 150 mm NaCl, 25 mm Tris-HCl, pH 7.6 and complete protease inhibitor cocktail (Roche, Basel, Switzerland)).

After lysis, cellular debris was removed using centrifugation at 12 000 g for 15 min, and the supernatant was collected. The supernatant was precleared by absorbing it with normal immunoglobulin G and 20 μl of protein-A or Protein-G sepharose beads for 1 h at 4 °C. The beads were removed using centrifugation, and the supernatant was subjected to immunoprecipitation by adding 1 μg rabbit polyclonal anti-Mieap antibody. The antibody mixtures were allowed to react on a rotating device overnight at 4 °C. Protein-A or Protein-G sepharose beads were added, and the mixtures were incubated for an additional 2 h at 4 °C. The beads were washed five times in cold lysis buffer, and the immune complexes were released from the beads by boiling in 2 × Laemmli sample buffer. Samples were loaded onto 10–15% SDS–polyacrylamide gel electrophoresis gels, and the electrophoresed proteins were subjected to western blot analysis using anti-Mieap antibody or anti-BNIP3 antibody.

### Immunocytochemistry and quantitative analysis of MALM

For immunocytochemistry, cells were seeded on eight-well chamber slides (5 × 10^4^ cells/well for LS174T, LoVo and each LS174T KD cells, and 1.5 × 10^4^ cells/well for HCT116) at 37 °C in conventional culture medium. For hypoxic exposure (2% O_2_), cells were placed in an airtight modulator incubator (TAITEC) with hypoxic gas (2% O_2_, 5% CO_2_ and 93% N_2_) for 72 h. Then, the cells were fixed in 2% paraformaldehyde for 10 min at room temperature. Slides were incubated with 0.1% Triton X-100 in phosphate-buffered saline (PBS) for 3 min and washed three times with PBS at room temperature. Cells were blocked with 3% bovine serum albumin in PBS for 1 h and sequentially incubated with primary antibody sets (anti-Mieap antibody (1:200)/anti-TOM20 antibody (1:200) or anti-LAMP1 antibody (1:200)/anti-TOM20 antibody (1:200)) for 1 h at room temperature. After washing three times with PBS, slides were incubated with fluorescein isothiocyanate-conjugated goat anti-rabbit immunoglobulin G antibody and Alexa Fluor 546 goat anti-mouse immunoglobulin G antibody for 1 h at room temperature. Slides were treated with 1 μm TO-PRO-3 (Invitrogen) for 15 min to stain the nuclei, and then washed four times with PBS. Slides were mounted with VECTASHIELD H-1000 (Vector Laboratories, Burlingame, CA, USA), and observed under an Olympus IX70 (Olympus, Tokyo, Japan) inverted fluorescence microscope coupled with a Radiance 2000 laser-scanning confocal system (Bio-Rad, Hercules, CA, USA).

MALM was evaluated as described previously.^2^ In brief, the overlapping of Mieap/mitochondrial signals or lysosome/mitochondrial signals (yellow) was analyzed by LuminaVision image analysis software (Visual System Division, Mitani Corporation, Tokyo, Japan) in 300–400 cells using the optimal threshold parameters. The yellow intensity was presented as the average of the calculated values per cell with error bars. The experiment was repeated three times and a similar result was obtained.

### Quantitative analysis of ROS

ROS generation by mitochondria in living cells was analyzed with the mitochondrial superoxide indicator dihydrorhodamine123 (DHR123; Sigma-Aldrich). The cells were seeded on eight-well chamber slides (5 × 10^4^ cells/well for LS174T control and each LS174T KD cells) at 37 °C in conventional culture medium. After 72 h of hypoxic culture condition (2% O_2_), the cells were incubated with 10 μm DHR123 for 10 min at 37 °C in serum-free media, washed twice with serum-free media and then incubated with 5 μm CellMask (Invitrogen) for 15 min to visualize the walls of live cells. After washing twice with serum-free media, stained cells were immediately observed under the confocal laser-scanning microscope, and the images were captured using an excitation filter of 543 nm and emission filter of 579 nm for DHR123. The intensity of DHR123 in each captured image was analyzed by LuminaVision image analysis software (Visual System Division, Mitani Corporation). The total areas of DHR123 intensity in 300–400 cells were extracted using the optimal threshold parameters and calculated with LuminaVision image analysis software. The DHR123 intensity was presented as the average of the calculated values per cell with error bars. The experiment was repeated three times and a similar result was obtained.

### Boyden chamber transwell migration and invasion assay

Transwell chamber with filter membrane of 8 μm pore size (Chemotaxicell, KURABO Industries, Osaka, Japan) was coated with Cell matrix Type I-C (Nitta Gelatin, Osaka, Japan) for collagen coating. For invasion assay, collagen-coated filter membrane was treated with 120 μl of Matrigel (BD Biosciences, San Jose, CA, USA; diluted 1:10 in conventional culture medium) for 2 h at 37 °C before cells were seeded. LS174T control and each KD cells were seeded on each chamber (1.5 × 10^5^ cells in 200 μl medium) and 600 μl of medium was added with or without 50 μm Ebselen into the bottom of a 24-well plate. After 48 h of hypoxic culture condition (2% O_2_) or normal culture condition (20% O_2_), cells that had migrated through the filter pores were fixed in 10% formalin for 30 min. Next, cells were stained with Crystal Violet solution (0.1% Crystal Violet, 2% ethanol, PBS) and non-migrating cells were gently removed from the upper chamber with cotton. Ten random fields were scanned, and the intensity of stained cells was extracted using the optimal threshold parameters and calculated with LuminaVision image analysis software. Intensity of Crystal Violet staining was presented as the average of the calculated values per field with error bars. The experiment was repeated three times and a similar result was obtained.

### Statistical analysis

The statistical significance between two groups of data sets was determined by Student's *t*-test (two-tailed) with *P*<0.01 regarded as statistically significant.

## Figures and Tables

**Table 1:** Summary of p53/Mieap/BNIP3 statuses in human colorectal cancers

*Case*	*Mieap methylated*	*BNIP3 methylated*	*p53 mutation*
5	+	−	ND
23	+	+	ND
25	+	+	ND
35	+	−	ND
55	+	−	ND
1	−	+	ND
6	−	+	ND
8	−	+	GGC→TGC (245G→C)
19	−	+	ND
20	−	+	ND
28	−	+	ND
30	−	+	ND
38	−	+	ND
40	−	+	ND
41	−	+	ND
43	−	+	ND
45	−	+	CGC→CAC (181R→H)
46	−	+	ND
49	−	+	ND
51	−	+	CGC→CAC (175R→H)
54	−	+	ND
58	−	+	CGT→CAT (273R→H)
4	−	−	ND
7	−	−	GAG→TAG (271E→stop)
18	−	−	ND
21	−	−	Deletion in Exon4
22	−	−	CGG→CAG (248R→Q)
29	−	−	ND
31	−	−	CCC→CTC (250P→L)
32	−	−	GCC→ACC (161A→T)
33	−	−	ND
34	−	−	ND
37	−	−	CGA→TGA (213R→stop)
39	−	−	CAC→CCC (168H→P)
42	−	−	ND
47	−	−	Deletion in Exon5 (12 bp→4 bp/stop)
48	−	−	CGG→CAG (248R→Q)
50	−	−	ND
57	−	−	ND

Abbreviation: ND, not detected.

## References

[bib1] 1Miyamoto Y, Kitamura N, Nakamura Y, Futamura M, Miyamoto T, Yoshida M et al. Possible existence of lysosome-like organella within mitochondria and its role in mitochondrial quality control. PLoS ONE 2011; 6: e16054. 21264221 10.1371/journal.pone.0016054 PMC3022026

[bib2] 2Kitamura N, Nakamura Y, Miyamoto Y, Miyamoto T, Kabu K, Yoshida M et al. Mieap, a p53-inducible protein, controls mitochondrial quality by repairing or eliminating unhealthy mitochondria. PLoS ONE 2011; 6: e16060. 21264228 10.1371/journal.pone.0016060 PMC3022033

[bib3] 3Nakamura Y, Kitamura N, Shinogi D, Yoshida M, Goda O, Murai R et al. BNIP3 and NIX mediate Mieap-induced accumulation of lysosomal proteins within mitochondria. PLoS ONE 2012; 7: e30767. 22292033 10.1371/journal.pone.0030767 PMC3266916

[bib4] 4Miyamoto T, Kitamura N, Ono M, Nakamura Y, Yoshida M, Kamino H et al. Identification of 14-3-3γ as a Mieap-interacting protein and its role in mitochondrial quality control. Sci Rep 2012; 2: 379. 22532927 10.1038/srep00379 PMC3334856

[bib5] 5Zhang J, Ney PA. Role of BNIP3 and NIX in cell death, autophagy, and mitophagy. Cell Death Differ 2009; 16: 939–946. 19229244 10.1038/cdd.2009.16 PMC2768230

[bib6] 6Bruick RK. Expression of the gene encoding the proapoptotic Nip3 protein is induced by hypoxia. Proc Natl Acad Sci USA 2000; 97: 9082–9087. 10922063 10.1073/pnas.97.16.9082 PMC16825

[bib7] 7Guo K, Searfoss G, Krolikowski D, Pagnoni M, Franks C, Clark K et al. Hypoxia induces the expression of the proapototic gene BNIP3. Cell Death Differ 2001; 8: 367–376. 11550088 10.1038/sj.cdd.4400810

[bib8] 8Sowter HM, Ratcliffe PJ, Watson P, Greenberg AH, Harris AL. HIF-1-dependent regulation of hypoxic induction of the cell death factors BNIP3 and NIX in human tumors. Cancer Res 2001; 61: 6669–6673. 11559532

[bib9] 9Vande Velde C, Cizeau J, Dubik D, Alimonti J, Brown T, Israels S et al. BNIP3 and genetic control of necrosis-like cell death through the mitochondrial permeability transition pore. Mol Cell Biol 2000; 20: 5454–5468. 10891486 10.1128/mcb.20.15.5454-5468.2000 PMC85997

[bib10] 10Cizeau J, Ray R, Chen G, Gietz RD, Greenberg AH. The *C. elegans* orthologue ceBNIP3 interacts with CED-9 and CED-3 but kills through a BH3- and caspase-independent mechanism. Oncogene 2000; 19: 5453–5463. 11114722 10.1038/sj.onc.1203929

[bib11] 11Dorn GW2nd. Mitochondrial pruning by Nix and BNip3: an essential function for cardiac-expressed death factors. J Cardiovasc Transl Res 2010; 3: 374–383. 20559783 10.1007/s12265-010-9174-x PMC2900478

[bib12] 12Tsuneki M, Nakamura Y, Kinjo T, Nakanishi R, Arakawa H. Mieap suppresses murine intestinal tumor via its mitochondrial quality control. Sci Rep 2015; 5: 12472. 26216032 10.1038/srep12472 PMC4516962

[bib13] 13Harris AL. Hypoxia—a key regulatory factor in tumour growth. Nat Rev Cancer 2002; 2: 38–47. 11902584 10.1038/nrc704

[bib14] 14Ishikawa K, Takenaga K, Akimoto M, Koshikawa N, Yamaguchi A, Imanishi H et al. ROS-generating mitochondrial DNA mutations can regulate tumor cell metastasis. Science 2008; 320: 661–664. 18388260 10.1126/science.1156906

[bib15] 15Murai M, Toyota M, Suzuki H, Satoh A, Sasaki Y, Akino K et al. Aberrant methylation and silencing of the BNIP3 gene in colorectal and gastric cancer. Clin Cancer Res 2005; 11: 1021–1027. 15709167

[bib16] 16Okami J, Simeone DM, Logsdon CD. Silencing of the hypoxia-inducible cell death protein BNIP3 in pancreatic cancer. Cancer Res 2004; 64: 5338–5346. 15289340 10.1158/0008-5472.CAN-04-0089

[bib17] 17Bacon AL, Fox S, Turley H, Harris AL. Selective silencing of the hypoxia-inducible factor 1 target gene BNIP3 by histone deacetylation and methylation in colorectal cancer. Oncogene 2007; 26: 132–141. 16799636 10.1038/sj.onc.1209761

[bib18] 18Abe T, Toyota M, Suzuki H, Murai M, Akino K, Ueno M et al. Upregulation of BNIP3 by 5-aza-2'-deoxycytidine sensitizes pancreatic cancer cells to hypoxia-mediated cell death. J Gastroenterol 2005; 40: 504–510. 15942716 10.1007/s00535-005-1576-1

[bib19] 19Kim JY, Cho JJ, Ha J, Park JH. The carboxy terminal C-tail of BNip3 is crucial in induction of mitochondrial permeability transition in isolated mitochondria. Arch Biochem Biophys 2002; 398: 147–152. 11831844 10.1006/abbi.2001.2673

[bib20] 20Bocharov EV, Pustovalova YE, Pavlov KV, Volynsky PE, Goncharuk MV, Ermolyuk YS et al. Unique dimeric structure of BNip3 transmembrane domain suggests membrane permeabilization as a cell death trigger. J Biol Chem 2007; 282: 16256–16266. 17412696 10.1074/jbc.M701745200

[bib21] 21Woo DK, Green PD, Santos JH, D'Souza AD, Walther Z, Martin WD et al. Mitochondrial genome instability and ROS enhance intestinal tumorigenesis in APC(Min/+) mice. Am J Pathol 2012; 180: 24–31. 22056359 10.1016/j.ajpath.2011.10.003 PMC3338350

[bib22] 22Szatrowski TP, Nathan CF. Production of large amount of hydrogen peroxide by human tumor cells. Cancer Res 1991; 51: 794–798. 1846317

[bib23] 23Toyokuni S, Okamoto K, Yodoi J, Hiai H. Persistent oxidative stress in cancer. FEBS Lett 1995; 358: 1–3. 7821417 10.1016/0014-5793(94)01368-b

[bib24] 24Nelson KK, Melendez JA. Mitochondrial redox control of matrix metalloproteinases. Free Radic Biol Med 2004; 37: 768–784. 15304253 10.1016/j.freeradbiomed.2004.06.008

[bib25] 25Klimova T, Chandel NS. Mitochondrial complex III regulates hypoxic activation of HIF. Cell Death Differ 2008; 15: 660–666. 18219320 10.1038/sj.cdd.4402307

[bib26] 26Guzy RD, Hoyos B, Robin E, Chen H, Liu L, Mansfield KD et al. Mitochondrial complex III is required for hypoxia-induced ROS production and cellular oxygen sensing. Cell Metab 2005; 1: 401–408. 16054089 10.1016/j.cmet.2005.05.001

[bib27] 27Lin X, David CA, Donnelly JB, Michaelides M, Chandel NS, Huang X et al. A chemical genomics screen highlights the essential role of mitochondria in HIF-1 regulation. Proc Natl Acad Sci USA 2008; 105: 174–179. 18172210 10.1073/pnas.0706585104 PMC2224181

[bib28] 28Schreck R, Rieber P, Baeuerle PA. Reactive oxygen intermediates as apparently widely used messengers in the activation of the NF-kappa B transcription factor and HIV-1. EMBO J 1991; 10: 2247–2258. 2065663 10.1002/j.1460-2075.1991.tb07761.x PMC452914

[bib29] 29Weinberg F, Chandel NS. Reactive oxygen species-dependent signaling regulates cancer. Cell Mol Life Sci 2009; 66: 3363–3673. 19629388 10.1007/s00018-009-0099-y PMC11115800

[bib30] 30Weinberg F, Hamanaka R, Wheaton WW, Weinberg S, Joseph J, Lopez M et al. Mitochondrial metabolism and ROS generation are essential for Kras-mediated tumorigenicity. Proc Natl Acad Sci USA 2010; 107: 8788–8793. 20421486 10.1073/pnas.1003428107 PMC2889315

[bib31] 31Sullivan LB, Chandel NS. Mitochondrial reactive oxygen species and cancer. Cancer Metab 2014; 2: 17. 25671107 10.1186/2049-3002-2-17 PMC4323058

[bib32] 32Schieber M, Chandel NS. ROS function in redox signaling and oxidative stress. Curr Biol 2014; 24: R453–R462. 24845678 10.1016/j.cub.2014.03.034 PMC4055301

[bib33] 33Giannoni E, Parri M, Chiarugi P. EMT and oxidative stress: a bidirectional interplay affecting tumor malignancy. Antioxid Redox Signal 2012; 16: 1248–1263 R. 21929373 10.1089/ars.2011.4280

[bib34] 34Radisky DC, Levy DD, Littlepage LE, Liu H, Nelson CM, Fata JE et al. Rac1b and reactive oxygen species mediate MMP-3-induced EMT and genomic instability. Nature 2005; 436: 123–127. 16001073 10.1038/nature03688 PMC2784913

[bib35] 35Cannito S, Novo E, Compagnone A, Valfre di Bonzo L, Busletta C, Zamara E et al. Redox mechanisms switch on hypoxia-dependent epithelial-mesenchymal transition in cancer cells. Carcinogenesis 2008; 29: 2267–2278. 18791199 10.1093/carcin/bgn216

