# Supplementary figures and images for: Mieap-regulated mitochondrial quality control is frequently inactivated in human colorectal cancer
Source: Oncogenesis. 2016 Jan 4;5(1):e181–. doi: 10.1038/oncsis.2015.43 (PMC4728673; doi:10.1038/oncsis.2015.43)

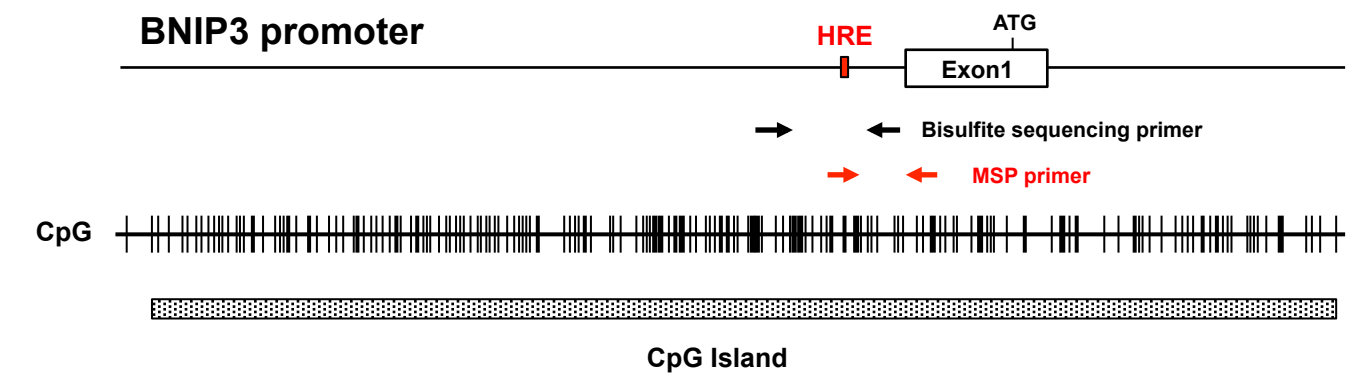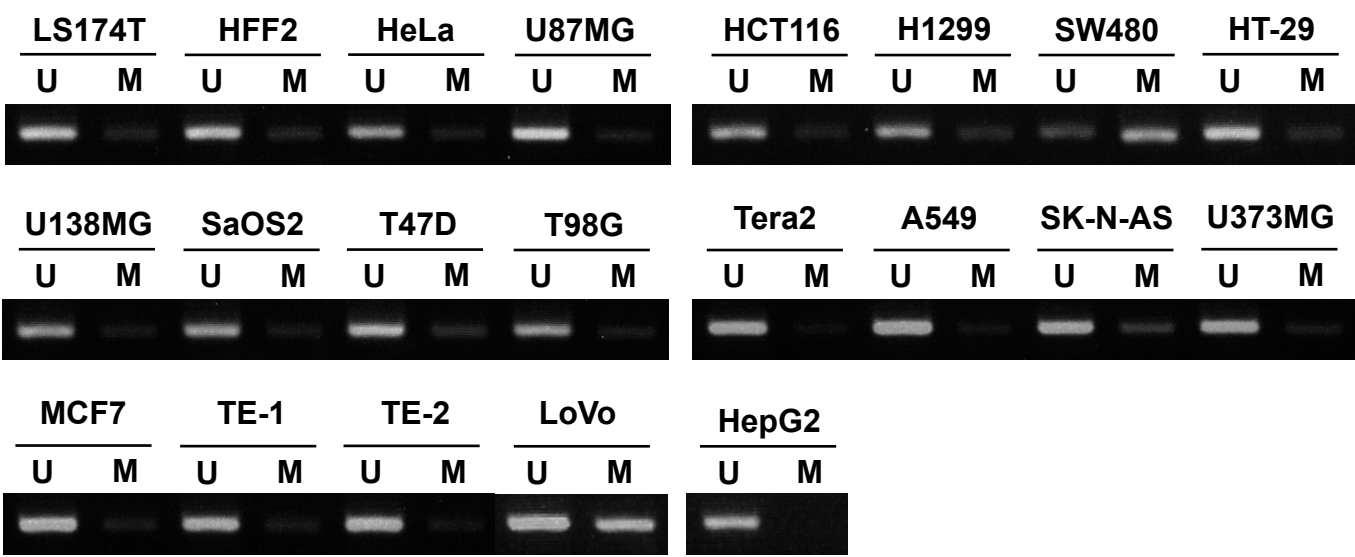

Supplement: Supplementary Figure 1 [file oncsis201543x1.pdf]

A

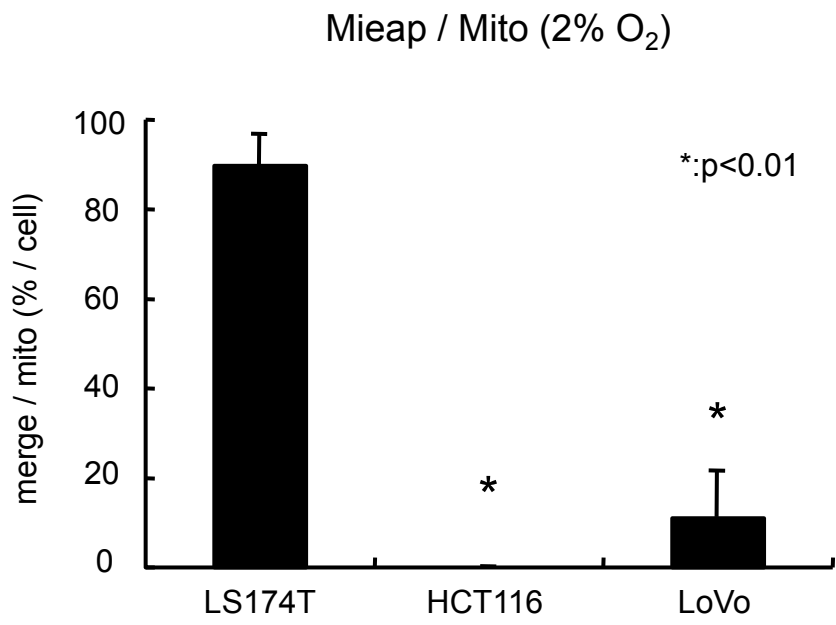

B

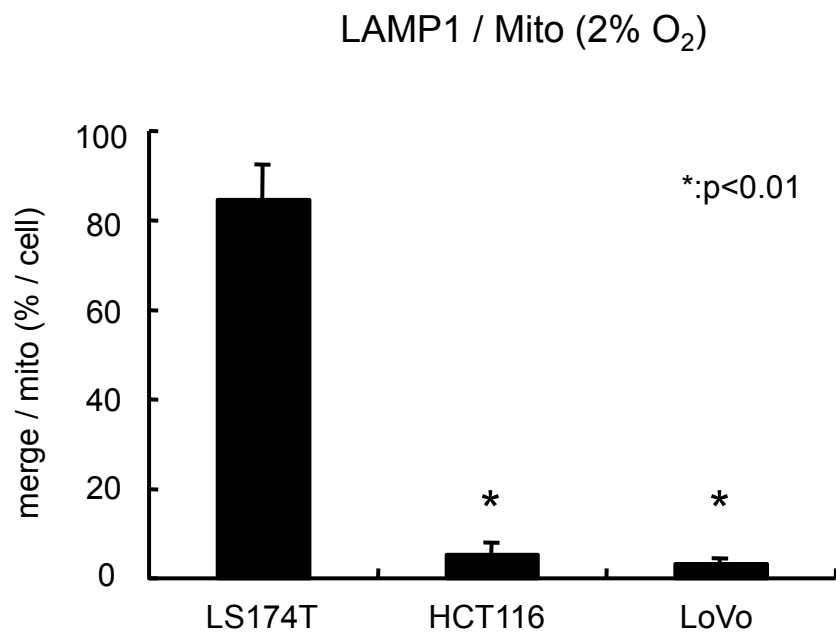

Supplement: Supplementary Figure 2 [file oncsis201543x2.pdf]
