# Supplementary material for: Mieap-regulated mitochondrial quality control is frequently inactivated in human colorectal cancer
Source: Oncogenesis. 2016 Jan 4;5(1):e181–. doi: 10.1038/oncsis.2015.43 (PMC4728673; doi:10.1038/oncsis.2015.43)
Supplement: Supplementary Figure Legends [file oncsis201543x3.pdf]

Supplementary Figure S1. MSP analysis of the BNIP3 promoter in various cancer cell lines.

(Upper) Primers for methylation specific PCR (MSP) and for bisulfite sequencing are located in the promoter region of BNIP3. HRE: hypoxia-response element. (Lower) MSP analysis of the BNIP3 promoter. Each genomic DNA was extracted from cultured cells, and used to bisulfite treatment. MSP was performed using MSP primer set for BNIP3. Almost all of cell lines showed un-methylation of BNIP3 promoter, but highly methylation in SW480 and half of methylation in LoVo was detected.

Supplementary Figure S2. Mieap-regulated mitochondrial quality control activity in three colorectal cancer cell lines including LS174T, HCT116, and Lovo, under hypoxia.

Quantitative analysis of the yellow or red areas of Figure 3C and 3D was carried out in 300-400 cells. Average values for the ratio of yellow to red signals (merged/mitochondrial; bar graph) Figure 3C and 3D are presented in A and B, respectively; the error bars indicate 1 SD. $p<0.01$ (*) was considered statistically significant.
