# Supplementary material for: Mieap-regulated mitochondrial quality control is frequently inactivated in human colorectal cancer
Source: Oncogenesis. 2016 Jan 4;5(1):e181–. doi: 10.1038/oncsis.2015.43 (PMC4728673; doi:10.1038/oncsis.2015.43)
Supplement: Supplementary Table 1 [file oncsis201543x4.doc]

**Supplementary Table S1. Mieap and BNIP3 promoter methylation statuses in colorectal tumor and normal samples of 57 colorectal cancer patients**

**Tumors** (total 57 case)

| Mieap / M, BNIP3 / M  Mieap / M, BNIP3 / UM | 2 case  3 case |
| --- | --- |
| Mieap / UM, BNIP3 / M | 26 case |
| Mieap / UM, BNIP3 / UM | 26 case |

Total 57 case

**Corresponded normal tissues** (total 57 case)

| Mieap / M, BNIP3 / M  Mieap / M, BNIP3 / UM | 0 case  0 case |
| --- | --- |
| Mieap / UM, BNIP3 / M | 0 case |
| Mieap / UM, BNIP3 / UM | 57 case |

Total 57 case
