# Supplementary material for: Mieap-regulated mitochondrial quality control is frequently inactivated in human colorectal cancer
Source: Oncogenesis. 2016 Jan 4;5(1):e181–. doi: 10.1038/oncsis.2015.43 (PMC4728673; doi:10.1038/oncsis.2015.43)
Supplement: Supplementary Table 2 [file oncsis201543x5.docx]

| **Supplementary Table S2. Summary of clinicopathological parameters in human colorectal cancers** | | | | | | | | |
| --- | --- | --- | --- | --- | --- | --- | --- | --- |
| **Case** | **Age** | **Gender** | **Site** | **Stage** | **TNM** | **Recurrence** | **5-year survival** | **Histopathological diagnosis** |
| 5 | 80 | Male | Rectum | IIA | T3N0M0 | - | yes | Moderately differentiated adenocarcinoma |
| 23 | 76 | Male | Rectum | I | T2N0M0 | - | yes | Moderately differentiated adenocarcinoma |
| 25 | 59 | Female | Colon | IV | T3N1M1 | + | yes | Moderately differentiated adenocarcinoma |
| 35 | 68 | Female | Rectum | IV | T3N0M1 | - | yes | Well differentiated adenocarcinoma |
| 55 | 82 | Female | Colon | IIA | T3N0M0 | - | no | Well differentiated adenocarcinoma |
| 1 | 66 | Male | Colon | IIA | T3N0M0 | - | yes | Well differentiated adenocarcinoma |
| 6 | 55 | Female | Rectum | IV | T3N2M1 | + | no | Poorly differentiated adenocarcinoma |
| 8 | 57 | Male | Rectum | IIIC | T3N2M0 | - | yes | Well differentiated adenocarcinoma |
| 19 | 77 | Male | Colon | IIA | T3N0M0 | - | unknown | Well differentiated adenocarcinoma |
| 20 | 52 | Male | Cecum | IV | T4N2M1 | - | no | Moderately differentiated adenocarcinoma |
| 28 | 67 | Female | Cecum | IIIB | T3N1M0 | - | yes | Well differentiated adenocarcinoma |
| 30 | 75 | Female | Rectum | IIIB | T2N2M0 | - | yes | Well differentiated adenocarcinoma |
| 38 | 70 | Male | Rectum | IIA | T3N0M0 | - | yes | Moderately differentiated adenocarcinoma |
| 40 | 56 | Male | Rectum | I | T1N0M0 | - | yes | Well differentiated adenocarcinoma |
| 41 | 50 | Male | Rectum | IIA | T3N0M0 | - | yes | Well differentiated adenocarcinoma |
| 43 | 75 | Female | Colon | IIA | T3N0M0 | - | yes | Well differentiated adenocarcinoma |
| 45 | 72 | Male | Colon | IV | T4N2M1 | - | no | Well differentiated adenocarcinoma |
| 46 | 61 | Male | Rectum | IIIB | T3N1M0 | + | yes | Well differentiated adenocarcinoma |
| 49 | 51 | Female | Colon | IV | T4N1M1 | - | yes | Well differentiated adenocarcinoma |
| 51 | 59 | Male | Rectum | IIIB | T3N2M0 | + | yes | Moderately differentiated adenocarcinoma |
| 54 | 74 | Female | Colon | IIA | T3N0M0 | - | unknown | Well differentiated adenocarcinoma |
| 58 | 74 | Male | Rectum | IIA | T3N0M0 | - | yes | Moderately differentiated adenocarcinoma |
| 4 | 69 | Male | Colon | IV | T4N1M1 | - | no | Moderately differentiated adenocarcinoma |
| 7 | 44 | Female | Colon | IIB | T4N0M0 | + | unknown | Well differentiated adenocarcinoma |
| 18 | 63 | Male | Rectum | IIIB | T3N2M0 | - | yes | Well differentiated adenocarcinoma |
| 21 | 49 | Male | Rectum | IIA | T3N0M0 | - | yes | Moderately differentiated adenocarcinoma |
| 22 | 63 | Female | Rectum | IV | T3N1M1 | - | yes | Moderately differentiated adenocarcinoma |
| 29 | 45 | Male | Rectum | IIA | T3N0M0 | - | yes | Well differentiated adenocarcinoma |
| 31 | 69 | Male | Rectum | IIA | T3N0M0 | - | yes | Moderately differentiated adenocarcinoma |
| 32 | 76 | Male | Rectum | IV | T4N2M1 | + | unknown | Moderately differentiated adenocarcinoma |
| 33 | 33 | Female | Colon | IIIB | T3N1M0 | - | yes | Moderately differentiated adenocarcinoma |
| 34 | 74 | Male | Rectum | IIA | T3N0M0 | - | no | Well differentiated adenocarcinoma |
| 37 | 74 | Male | Colon | IV | T3N2M1 | - | no | Moderately differentiated adenocarcinoma |
| 39 | 64 | Male | Rectum | IV | T3N2M1 | + | no | Poorly differentiated adenocarcinoma |
| 42 | 70 | Male | Rectum | IIA | T3N0M0 | + | no | Moderately differentiated adenocarcinoma |
| 47 | 56 | Female | Colon | IIA | T3N0M0 | - | unknown | Well differentiated adenocarcinoma |
| 48 | 62 | Female | Rectum | IIIB | T3N2M0 | + | yes | Well differentiated adenocarcinoma |
| 50 | 75 | Male | Cecum | IIIC | T4N1M0 | - | no | Well differentiated adenocarcinoma |
| 57 | 33 | Male | Rectum | IIA | T3N0M0 | - | unknown | Mucinous adenocarcinoma |
