# Supplementary material for: Mieap-regulated mitochondrial quality control is frequently inactivated in human colorectal cancer
Source: Oncogenesis. 2016 Jan 4;5(1):e181–. doi: 10.1038/oncsis.2015.43 (PMC4728673; doi:10.1038/oncsis.2015.43)
Supplement: Supplementary Materials and Methods [file oncsis201543x6.pdf]

Supplementary Materials and Methods

p53 mutation search by using direct sequencing method

Genomic DNA samples from primary colon tumors and corresponding normal colorectal tissues were analyzed for the presence of p53 mutations in exon 4 to 8. The primers used for PCR amplification were as follows: forward, 5'-GAC TGC TCT TTT CAC CCA TC-3' and reverse, 5'-CCA AAG GGT GAA GAG GAA TC-3' for exon 4; forward, 5'-GTT TCT TTG CTG CCG TCT TC-3' and reverse, 5'-GCA ATC AGT GAG GAA TCA GAG-3' for exon 5; forward, 5'-CCT CTG ATT CCT CAC TGA TTG-3' and reverse, 5'-TCA TGG GGT TAT AGG GAG GTC-3' for exon 6; forward, 5'-TCT TGG GCC TGT GTT ATC TC-3' and reverse, 5'-TGG ATG GGT AGT AGT ATG GAA G-3' for exon 7; forward, 5'-ACA AGG GTG GTT GGG AGT AG-3' and reverse, 5'-GGA AAG AGG CAA GGA AAG GTG-3' for exon 8. PCR reaction was carried out using following conditions: 1 cycle at 94°C for 5 min, 30 cycle at 94°C for 30 s, 55°C for 30 s, and 72°C for 30 s, and final extension step at 72°C for 4 min. PCR products were purified by standard EtOH precipitation methods, and used for sequence reaction PCR with each forward or reverse primer. Samples were sequenced with ABI 3100 genetic analyser (Applied Biosystems).

Establishment of BNIP3-KD cell lines using RNA interference

We have already established p53-KD,[36] Mieap-KD[1] and experimental control cells[1] using LS174T cell line, as described previously.[1,36] Here, we established BNIP3-KD LS174T cells using methods as described previously.[36] BNIP3 expression was inhibited in LS174T cells by retroviral expression of short-hairpin RNA (shRNA) (BNIP3-KD: 5'-gatccccACACGAGCGTCATGAAGAAttcaagagaTTCTTCATGACGCTCGTGTttttt ggaaa-3') against the BNIP3 sequence.

Immunoblotting

LS174T control, Mieap-KD and BNIP3-KD cells were seeded (2×10^6 cells / 6 cm-dish) at 37°C in conventional culture medium. Next day, cells were placed in an airtight modulator incubator with hypoxic gas (2% O_2, 5% CO_2 and 93% N_2) or normoxic gas (20% O_2, and 5% CO_2) and incubated for 48 h. Then, cells were collected, lysed in Urea-SDS buffer (6.7M Urea, 1% SDS, 10mM Tris-HCl pH6.8, 1mM DTT, 10% glycerol, Protease Inhibitor cocktail) and sonicated on ice. Homogenates were

centrifuged for 5 min in a micro- centrifuge at 4°C and supernatants were collected and boiled in 5×SDS sample buffer (125mM Tris-HCl pH6.8, 4% SDS, 20% glycerol, 10% 2-mercaptoethanol and 0.4mg/ml bromophenol blue). Each sample (15μg) was loaded onto a 12% SDS-poly-acrylamide gel electrophoresis (PAGE) gel and blotted onto a nitrocellulose membrane (GE Healthcare). Western blots were performed with each primary antibody (anti-BNIP3 antibody; 2μg/ml or anti-Mieap antibody; 1:1000 dilution). Protein bands on the membrane were visualized using ECL Plus Western Blotting detection reagent (GE Healthcare).

36. Masuda Y, Futamura M, Kamino H, Nakamura Y, Kitamura N, Ohnishi S, et al. The potential role of DFNA5, a hearing impairment gene, in p53-mediated cellular responses to DNA damage. *J Hum Genet* 2006; **51**: 652-664.
